# Risk of suicidal acts in patients with dissociative disorder: a population-based cohort study

**DOI:** 10.1186/s12991-025-00622-5

**Published:** 2026-02-03

**Authors:** Wai Kwong Tang, Kelvin K. F. Tsoi, Terry Cheuk Fung Yip, Vivien Wei Jun Liew, Selina Kit Yi Chan

**Affiliations:** 1 Department of Psychiatry, The Chinese University of Hong Kong, Shatin, New Territories, Hong Kong SAR China; 2 Jockey Club School of Public Health and Primary Care, The Chinese University of Hong Kong, Shatin, New Territories, Hong Kong SAR China; 3 Stanley Ho Big Data Decision Analytics Research Centre, The Chinese University of Hong Kong, Shatin, New Territories, Hong Kong SAR China; 4 Department of Medicine and Therapeutics, The Chinese University of Hong Kong, Shatin, New Territories, Hong Kong SAR China

**Keywords:** Dissociative disorder, Self-harm, Risk, Depression, Suicidal, Cohort study

## Abstract

**Background:**

This study aimed to examine if individuals diagnosed with dissociative disorder (DD) are at a higher risk of suicidal acts compared to individuals without DD.

**Methods:**

We conducted a matched cohort study by examining electronic health records of patients admitted to Hong Kong public hospitals between January 1, 1993, through December 31, 2022. The study included 716 patients with DD and a matched comparison cohort of 716 individuals. Participants were tracked until they either received a diagnosis of suicidal acts, died from other causes, or until the end of 2023, whichever happened first. Cox proportional hazards regression models were applied to determine the risk of suicidal acts following DD onset.

**Results:**

During the 30-year study period, 87 individuals (12.2%) in the DD cohort and 29 individuals (4.1%) in the comparison cohort exhibited suicidal acts, with the difference being statistically significant (χ^2^ = 25.21, *p* = 0.0001). The incidence rates of suicidal acts were 86.7 and 27.3 per 10,000 person-years for the DD and comparison cohorts, respectively. After adjustment, the hazard ratio for suicidal acts in the DD cohort compared with the comparison cohort was 1.89 (95% confidence interval, 1.17–3.06).

**Conclusions:**

DD is linked to a heightened risk of suicidal acts. Future research is required to replicate these findings and to more comprehensively identify the specific risk factors contributing to suicidal acts in this patient population.

## Background

Dissociative disorder (DD) is characterized by a disruption of and/or discontinuity in the normal integration of consciousness, memory, identity, emotion, perception, body representation, motor control, and behavior [1]. DD encompasses dissociative identity disorder, dissociative amnesia, depersonalization/derealization disorder, other specified DD, and unspecified DD. The prevalence of different subtypes of DD among women ranges from 0.2% (dissociative fugue) to 8.3% (DD not otherwise specified) [2].

DD is frequently associated with poor self-esteem, depression, daily functional impairment, higher levels of disability, and overall poor quality of life [3–5]. Comorbid major depressive disorder is common in people with DD [6]. Given the negative consequences associated with DD and the elevated risk of co-occurring major depressive disorder, the rate of suicidal attempt is also likely to be elevated in this population.

DD patients have been found to engage in more frequent suicide attempts (SA) than patients with other psychiatric disorders [7]. A study of 241 patients with DD showed that dissociative symptoms were higher among those with a history of suicidal attempt than those without [8]. Another study that investigated the relationships between emotion dysregulation, dissociation, and self-injury reported that 69% of DD patients had a history of self-injury [9]. In a study of 50 DD patients, 82% reported self-mutilation behaviours [10]. A study of 200 male substance-dependent inpatients reported a larger proportion of suicide attempts and self-mutilation in the dissociative group than the non-dissociative group [11]. A meta-analysis of 11 studies (*n* = 382) suggested that the presence of a DD diagnosis was related to suicidal attempts in psychiatric patients (odds ratio = 6 (range 2 to 16) [12]. One literature review concluded that DD is consistently associated with increased suicidal attempts [13]. The limitations of the original studies included a cross-sectional design [9]; recruitment of selective samples (male, white, treatment-seeking subjects, inpatients, comorbid drug dependency, access to internet) [9, 11]; small sample size [10]; use of dissociative symptoms rather than a formal diagnosis of DD as the independent variable [8]; use of self-injury or self-mutilation instead of suicidal attempt as the outcome [9, 10]; and use of a non-validated tool to assess the outcome [9]. Limitations of the review and meta-analyses studies include a lack of access to articles [12], limited numbers of studies [12], and unknown direction of association [12]. The purpose of present study was to determine whether individuals with DD are at increased risk of suicidal acts relative to those without DD.

## Methods

### Data source

Using the Hong Kong Clinical Data Analysis and Reporting System (CDARS), we examined the electronic health records of every patient admitted to Hong Kong public hospitals for any indication between January 1, 1993, and December 31, 2022, as part of this matched cohort study. Data from the CDARS have been employed in prior epidemiological research and have demonstrated proven reliability [14]. This system comprises electronic health records maintained by the Hong Kong Hospital Authority, a statutory organization responsible for the administration of all public hospitals serving Hong Kong’s 7.7 million residents. The healthcare framework in Hong Kong currently delivers three tiers of medical services across both public and private sectors: primary, secondary, and tertiary care [15]. Public healthcare services in Hong Kong are overseen by the Hospital Authority, which manages all public hospitals. Data from 2017 revealed that the Hospital Authority was responsible for approximately 80% of inpatient visits and 30% of outpatient visits [16]. To ensure patient privacy, the CDARS encrypts personal information and assigns anonymous identification numbers to individuals. The validity and reliability of the CDARS database have been established; for example, a validation study reported a positive predictive value of 96.8% for fracture diagnosis recorded within the system [17]. The institutional review board of the Chinese University of Hong Kong granted approval for this study (CREC 2020.707).

### DD cohort

Cases were defined as people who received outpatient, emergency department, or inpatient care with a first recorded diagnosis of DD during the study period. The date of the initial DD diagnosis was designated as the start of follow-up for each case. The identification of DD was based on the International Classification of Diseases (ICD)−9 codes 312.12 through 321.15.

### Non-DD comparison cohort

To establish a comparable non-DD cohort, we randomly identified individuals with no prior DD diagnosis, matching them to DD cases by age and sex at admission. The matching procedure was conducted at the individual level. Each control was matched to a case based on gender, age, and the number of years since being diagnosed with DD. A random month and day within the same index year as the matched patient’s index date were assigned. The matched admission date served as the start of follow-up for all comparators.

### Covariates

For both the DD and comparison cohorts, data on ethnicity, residential district, and diagnoses of depression and bipolar disorders (ICD 9 code 296) were also obtained from the CDARS database.

### Outcome measurement

Considering that hospital administrative datasets tend to under-report suicidal acts due to stigma and difficulties in establishing intent [18], we utilized an expanded definition of suicidal acts than the conventional ICD-9 criteria to encompass all self-injurious behaviors, both unintentional and intentional, as well as with or without suicidal intent. Suicidal acts were identified using ICD-9 codes E950-59 (self-harm) and E980-89 (self-harm of undetermined intent). Both cases and matched comparators were followed from the date of DD diagnosis (or the matched admission date for comparators) until the earliest occurrence of suicidal acts, death from any cause, or December 31, 2023, whichever happened first.

### Statistical analysis

For both DD cases and comparators, the count and proportion of participants who exhibited suicidal acts were calculated. The Chi-square (χ^2^) test was employed to examine differences in the proportions of suicidal acts between the two cohorts. Kaplan–Meier method was utilized to plot incidence curves, so that the temporal patterns of suicidal acts following the initial diagnosis of DD can be illustrated. The level of statistical significance was determined using the log-rank test. The incidence of suicidal acts was calculated as the number of suicidal acts divided by the total follow-up time, expressed per 10,000 person-years. Cox proportional hazards regression models were applied to estimate the hazard ratio (HR) and the 95% confidence interval (CI) for the risk of suicidal acts from the onset of DD, accounting for time at risk and adjusting for age, sex, ethnicity, residential district, depression, and bipolar disorders. All statistical analyses were performed using STATA (StataCorp, College Station, TX, USA) for all statistical analyses. The possible effect of COIVD-19 was also examined. We compared the risk of suicidal acts between subjects recruited during COVID-19 (February 2020 to April 2023) [19] and before COVID-19 (October 2016 to January 2020) in the DD group. The difference in the number of subjects with suicidal acts was analyzed using chi-square test, whereas the total number of episodes of suicidal acts was analyzed using the zero-inflated negative binomial regression. A *p* value of < 0.05 was considered statistically significant.

## Results

**Fig. 1:**
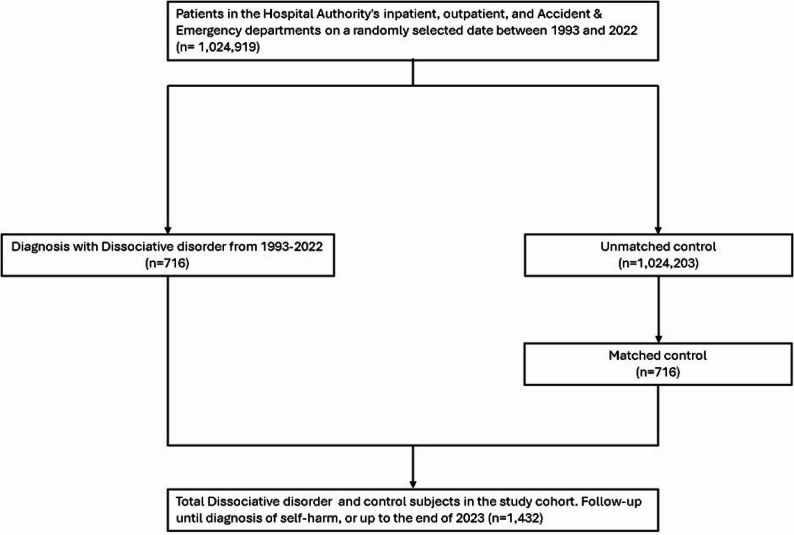
The flowchart of participant selection

**Fig. 2:**
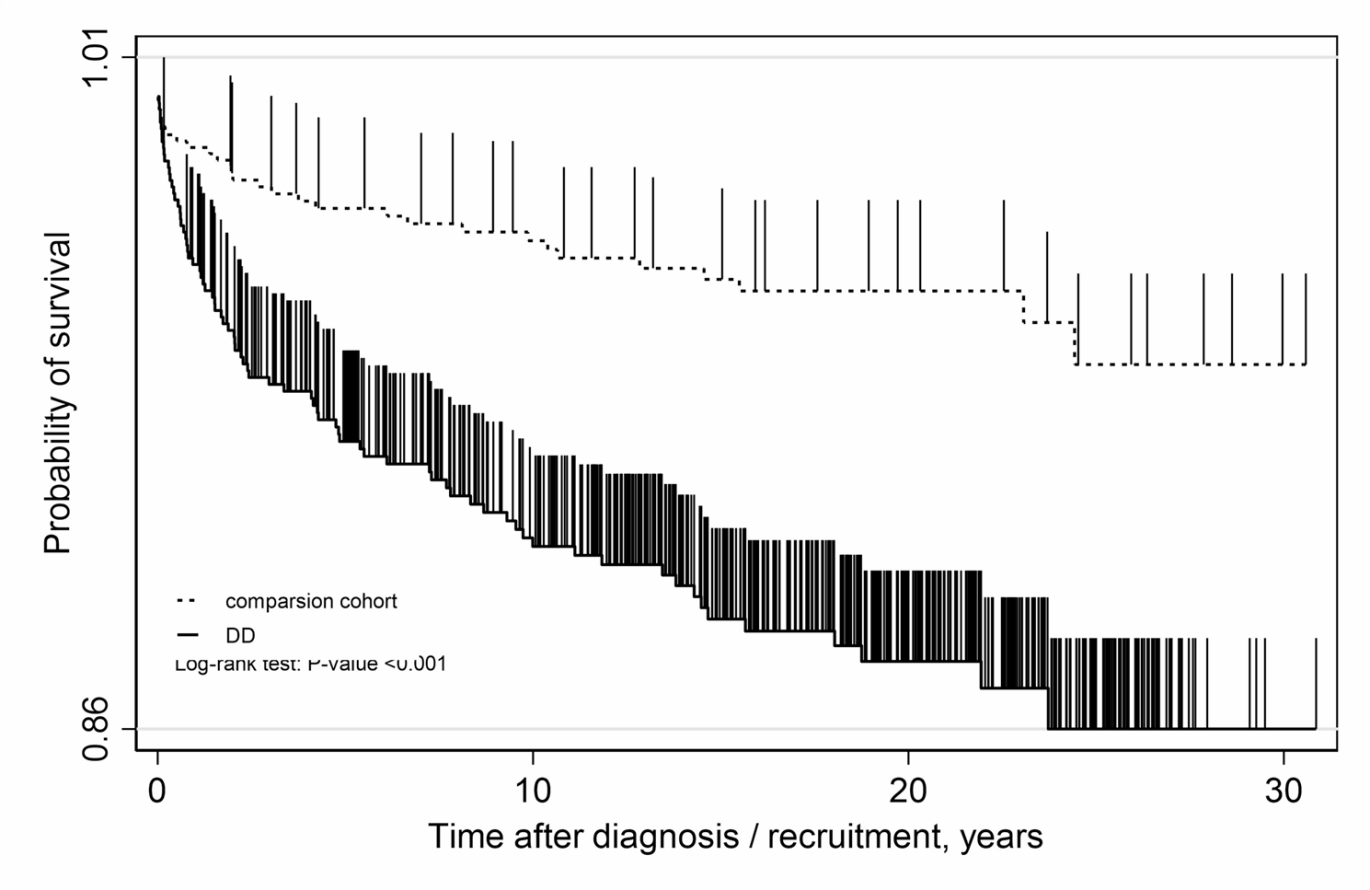
Kaplan-Meier survival estimates plot between DD group and comparison cohort

A DD cohort consisting of 716 patients and a matched comparison cohort of 716 subjects were selected (Fig. 1). There were no significant differences between the DD and comparison cohort in terms of sex distribution (31.28% female), ethnicity, or age (37.1 ± 16.9 years). Participants in the DD cohort were more frequently residents of Kowloon and the New Territories. The mean follow-up duration for the DD group was 14.1 years (SD 8.2), with a median of 14.6 years (IQR 7.0–20.9). For the control group, the mean was 14.9 years (SD 7.9), with a median of 15.9 years (IQR 7.8–20.2). The prevalence of depression and bipolar disorders was higher in the DD cohort (25.0%) relative to the comparison cohort (3.6%) (Table 1). Over the 30-year study period, the incidence of suicidal acts was 86.7 per 10,000 person-years in the DD cohort and 27.3 per 10,000 person-years in the comparison cohort. Kaplan–Meier analysis demonstrated a significant difference in suicidal acts incidence between the DD and comparison cohorts (log-rank *p* = 0.0001; Fig. 2). The unadjusted HR for suicidal acts in the DD cohort relative to the comparison cohort was 2.88 (95% CI, 1.87–4.44, *p*-value < 0.001). After adjusting for age, sex, ethnicity, residential district, and comorbid depression and bipolar disorders, the HR was 1.89 (95% CI, 1.17–3.06, p-value = 0.009). The mean time to suicidal acts 4.3 ± 5.6 years in the DD cohort and 5.7 ± 6.7 years in the comparison cohort. The median (interquartile range) time to suicidal acts was 1.6 (IQR: 0.3,7.2) in DD group, and 2.7 (IOR: 0.4,10.1) in the comparison cohort. In terms of the effect of COVID-19, amongst subjects recruited till September 2016 (*n* = 610), there was no significant difference in number of subjects with suicidal acts during and before and during COVID-19 (*n* = 15 and 11, *p* = 0.427). Similarly, there was no significant difference between the total number of episodes of suicidal acts during and before and during COVID-19 (*n* = 55 and 29, *p* = 0.518).

**Table 1:** Demographic and clinical characteristics in the study cohort

	DD (*n* = 716)	Comparison cohort (*n* = 716)	*p* value ^a^
Gender			
Female Male	224 (31.3) 492 (68.7)	224 (31.3) 492 (68.7)	1.000
Age (mean ± standard deviation)	37.1 ± 16.9	37.1 ± 16.9	1.000
Ethnicity ^c^			
Chinese Non-Chinese	649 (90.6) 43 (6.0)	608 (84.9) 31 (4.3)	0.278
District of residence ^d^			
Kowloon Hong Kong Island New Territories	296 (41.3) 143 (20.0) 276 (38.5)	213 (29.7) 262 (36.6) 166 (23.2)	0.004 < 0.001 < 0.001
History of depression/bipolar disorder	179 (25.0)	26 (3.6)	< 0.001
Time to suicidal acts (years), mean ± SD, median (IQR)	4.3 ± 5.6 1.6 (0.3–7.2)	5.7 ± 6.7 2.7 (0.4–10.1)	0.303

^a^ Pearson chi-square test; ^b^ T-test; ^c^ 24 and 77 subjects had no ethnicity data in DD and comparison group, respectively, ^d^1 and 75 subject had no district of residence data in DD and comparison cohort respectively

SD = standard deviation; IQR = interquartile range

## Discussion

This study is, to our knowledge, the first to methodically assess the risk of suicidal acts in patients subsequent to their initial diagnosis of DD. The patients had an elevated risk of suicidal acts, even after adjusting for diagnosed mood disorders, with an HR of 1.95. As far as we are aware, no previous study has reported the HR for suicidal acts in patients with DD. Our figure was smaller than the odds ratio of 6 reported in a meta-analysis [12]. In the present study, 12.2% of patients inflicted suicidal acts. In a meta-analysis of 11 studies, the rate of suicidal attempt ranged from 29% to 88%, with an average of 57% [12]. Possible explanations for the lower figures in the present study include under-reporting of suicidal acts in the DD group, an elevated risk of suicidal acts in the comparison group, and a relatively low rate of mood disorders in the DD group. There are also methodological differences between our study and Calati’s [12]; namely, their study was a meta-analysis rather than an original study; they included studies with small sample sizes and studies focusing on special groups, such as drug dependency, gender dysphoria, bipolar disorder, and major depression, and studies using self-report as a measure of suicidal acts; and their analysis did not adjust for age, gender, ethnicity or depression/bipolar disorder.

There are several limitations in the current study. The sample was derived from medical records within the CDARS database, excluding data from private hospitals, clinics, and general outpatient clinic services. While the study incorporated a well-matched comparison cohort based on age and sex, the comparators selected from hospital and outpatient clinic records likely possessed a higher baseline risk of suicidal acts than the general population, which may have resulted in an underestimation of the HRs.

We adopted a broad definition of suicidal acts encompassing deliberate self-injurious behaviors with a suicidal intent, self-injurious acts without suicidal intent, as well as both intentional and unintentional self-injurious actions. In a Hong Kong study examining self-harm risk among psychiatric patients, sensitivity analyses demonstrated that excluding cases of undetermined self-harm (ICD-9 codes E980–89) did not significantly alter the findings [20]. Furthermore, our dataset lacked information on interventions and treatments received by patients, which could have impacted the outcomes. It is important to note that the CDARS database records only suicidal acts, while the prevalence of suicidal ideation likely exceeds the documented frequency of suicidal acts and may be under-reported in clinical settings.

Our objective was to investigate the relationship between the onset of DD and subsequent suicidal acts. However, since clinical records prior to 1993 were unavailable, we could not confirm whether the earliest recorded diagnosis in the database truly represented the initial onset of DD. Additionally, suicidal acts data were derived solely from medical records, which may carry a risk of under-reporting.

Lastly, suicidal acts are complex behaviors influenced by a variety of factors, including demographic, social, economic, cultural, psychological, and environmental components [21]. However, due to limitations in our data source, we were unable to account for these risk factors. Ideally, information regarding sociodemographic variables such as marital and employment status, education level, as well as potential confounders like physical comorbidities, smoking, and alcohol use, should be available and adjusted for in the analysis.

## Conclusions

DD is linked to a heightened risk of suicidal acts. Future research is needed to replicate our findings and to more comprehensively identify the potential unique risk factors for suicidal acts in this patient population.

## Data Availability

The data used in this study were obtained from the Clinical Data Analysis and Reporting System (CDARS) managed by the Hospital Authority (HA) in Hong Kong. Due to privacy and legal restrictions, data access is strictly controlled and limited to anonymized datasets, subject to HA approval. Data request must be made through the HA’s official channels. Requests to access these datasets should be directed to Hospital Authority at https://www3.ha.org.hk/data/Provision/ApplicationProcedure.

## Abbreviations

DD: Dissociative Disorder
ICD-9: International Classification of Diseases
HR: Hazard Ratio
CI: Confidence Interval
